# The causal relationship between bacterial pneumonia and diabetes: a two-sample mendelian randomization study

**DOI:** 10.1080/19382014.2023.2291885

**Published:** 2023-12-14

**Authors:** Songying Pan, Zhongqi Zhang, Weiyi Pang

**Affiliations:** a The School of Public Health, Guilin Medical University, Guilin, Guangxi, China; b Guangxi Key Laboratory of Environmental Exposomics and Entire Lifecycle Health, Guilin Medical University, Guilin, Guangxi, China

**Keywords:** Bacterial pneumonia, causal relationship, diabetes, GDM, Mendelian randomization study, T1DM

## Abstract

The escalating worldwide occurrence of diabetes mellitus, recognized as a chronic metabolic ailment contributing to an amplified global disease burden, has stimulated researchers to explore its etiology. Consequently, the study employed a two-sample Mendelian randomization (MR) methodology to examine the causal connection between bacterial pneumonia and diabetes, drawing upon the existing observational study that identified a potential association between bacterial pneumonia and diabetes. Furthermore, MR investigations suggest a reciprocal causal relationship between bacterial pneumonia and gestational diabetes mellitus(GDM), and a plausible causal link between bacterial pneumonia and T1DM.

**Background:**

Previous observational studies have established the high prevalence of bacterial pneumonia in diabetic patients, which in turn leads to increased mortality. However, the presence of a causal connection between bacterial pneumonia and diabetes remains unobserved.

**Methods:**

We chose genome-wide significant (Ρ < 1 × 10^−5^ and Ρ < 1 × 10^−6^) and independent (r^2^ < 0.001) single-nucleotide polymorphisms (SNPs) as instrumental variables (IVs) to proceed a bidirectional two-sample MR study. The extracted SNPs explored the relationship between bacterial pneumonia and diabetes by Inverse variance weighted (IVW), MR-Egger, and weighted median methods. In addition, we conducted the Heterogeneity test, the Pleiotropy test, MR-presso and the Leave-one-out (LOO) sensitivity test to validate the reliability of results.

**Results:**

In an MR study with bacterial pneumonia as an exposure factor, four different types of diabetes as outcome. It was observed that bacterial pneumonia increases the incidence of GDM (OR = 1.150 (1.027–1.274, *P* = 0.011) and T1DM (OR = 1.277 (1.024–1.531), *P* = 0.016). In the reverse MR analysis, it was observed that GDM (OR = 1.112 (1.023–1.201, *P* = 0.009) is associated with an elevated risk of bacterial pneumonia. However, no significant association was observed bacterial pneumonia with T1DM and other types of diabetes (*P* > 0.05).

**Conclusion:**

This study utilizing MR methodology yields robust evidence supporting a bidirectional causal association between bacterial pneumonia and GDM. Furthermore, our findings suggest a plausible causal link between bacterial pneumonia and T1DM.

## Graphical abstract

**Figure:**
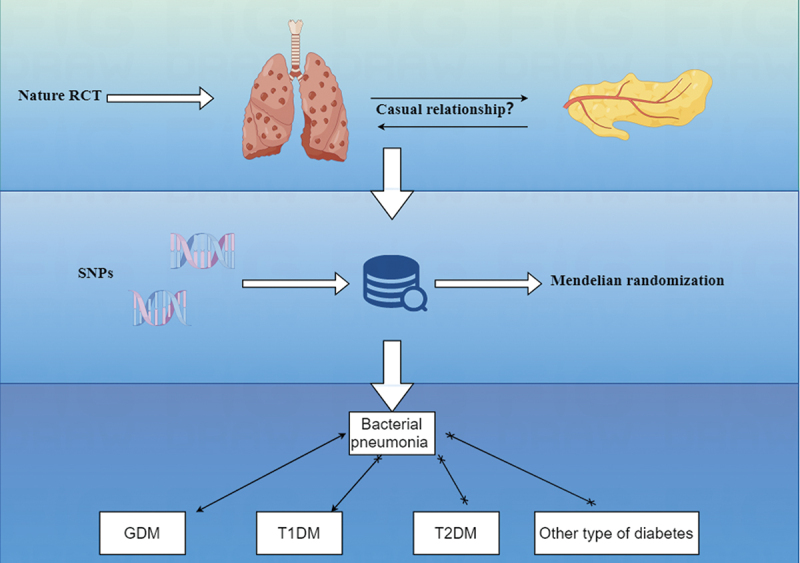

## Introduction

Diabetes mellitus, characterized primarily by hyperglycemia and resulting in significant metabolic disturbances, poses challenges in early detection. However, timely screening and intervention can effectively mitigate complications and manage the condition. According to the International Diabetes Federation, projections indicate a staggering 537 million individuals will be affected by diabetes in 2021, with a potential increase of 19.7% by 2030. Consequently, it is anticipated that over 10% of the global population will be afflicted by diabetes in the coming years.^1^ This chronic ailment, characterized by a significant disease burden, can be categorized into type 1 diabetes mellitus (T1DM), type 2 diabetes mellitus (T2DM), GDM, and other types of diabetes.^2^

T1DM arises from the autoimmune-mediated impairment of insulin-producing beta cells, leading to diminished or absent insulin secretion. Additionally, scholarly investigations have posited that the inability of beta cells to undergo regeneration constitutes the fundamental etiology of T1DM.^3^ T1DM, being a prototypical polygenic genetic disorder, is presently postulated to involve the involvement of determinants located at the DRB1, DQA1, and DQB1 loci on chromosome 6p21.^4^ Furthermore, it was observed that the abundance of *phylum bacteroidetes* was elevated in individuals diagnosed with T1DM,^5^ and the administration of probiotics led to a reduction in insulin needs among children with T1DM.^6^ These findings suggest a potential association between bacterial presence and the pathogenesis of T1DM.

Despite the presence of shared symptoms between T2DM and T1DM, the pathogenesis of T2DM distinguishes itself by exhibiting cellular insensitivity to insulin and inadequate insulin production by beta cells, resulting in an inability to meet the body’s demand through feedback regulation.^1^ The etiology of type 2 diabetes mellitus (T2DM) is characterized by a heightened complexity, owing to the robust genetic correlation between the MTNR1B rs10830963 G allele and T2DM.^7^ In addition, several research studies have revealed that the salivary microorganisms *Firmicutes*, *Lactobacillus*, *Veillonela*, and *Tannerella/T. forsythia* are enriched in patients with T2DM.^8^ obesity,^9^ and cardiovascular disease.^10^ Consequently, it can be inferred that the development of T2DM is influenced not only by genetic factors but also by external bacterial.

Glucose intolerance during pregnancy is commonly identified as gestational diabetes mellitus (GDM).^11^ While GDM typically resolves postpartum, it elevates the risk of developing type 2 diabetes mellitus (T2DM) after delivery.^12^ Furthermore, the emergence of GDM is linked to factors, such as age, body weight, polycystic ovary syndrome, and a familial predisposition to diabetes.^13^

Other types of diabetes refer to specific types of diabetes that are caused by various factors, such as monogenic diabetes syndromes, diseases affecting the exocrine pancreas, and diabetes induced by drugs or chemicals.^2^

The development of diabetes is commonly associated with both genetic factors and the external environment. It has been observed that individuals with diabetes who also have a K. pneumoniae infection experience a higher occurrence of sepsis and invasive infections. Furthermore, K. pneumoniae has been found to thrive in high glucose environments, resulting in elevated expression of the rmpA and ompA genes in hvKP. Consequently, this upregulation contributes to enhanced resistance against the immune system via the cAMP signaling pathway.^14^ These observational studies provide preliminary evidence on potential exposure factors associated with bacterial pneumonia in individuals with diabetes. In previous observational studies, the presence of reverse causality has posed a challenge, rendering it arduous to ascertain a causal association between bacterial pneumonia and diabetes. However, the MR research approach employs genetic variation as an instrumental variable, wherein the allocation of genes to individuals occurs randomly before birth, thereby circumventing the influence of confounding factors and reverse causality. Stated differently, the application of MR in observational studies entails utilizing natural randomized controlled trials to infer causality between exposure and outcome. Therefore, MR research serves as a crucial approach for establishing causal inference when clinical randomized controlled trials (RCTs) are lacking. Consequently, we have opted to undertake a bidirectional two-sample MR study to explore the causal relationship between diabetes mellitus and bacterial pneumonia.

## Materials and methods

**Figure 1.**
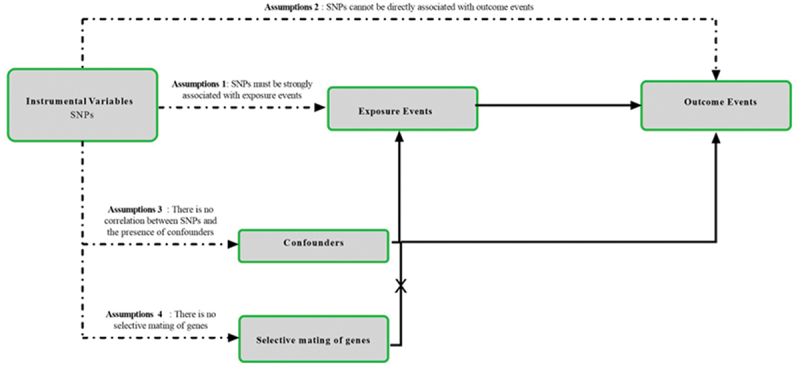
Research flow chart. MR test needs to meet four assumptions at the same. time. First, SNPs must be strongly correlated with exposure. Second, SNPs cannot be directly related to outcome. Third, SNPs cannot be associated with any possible confusing factors. Last, no genetic assortative mating.

**Figure 2.**
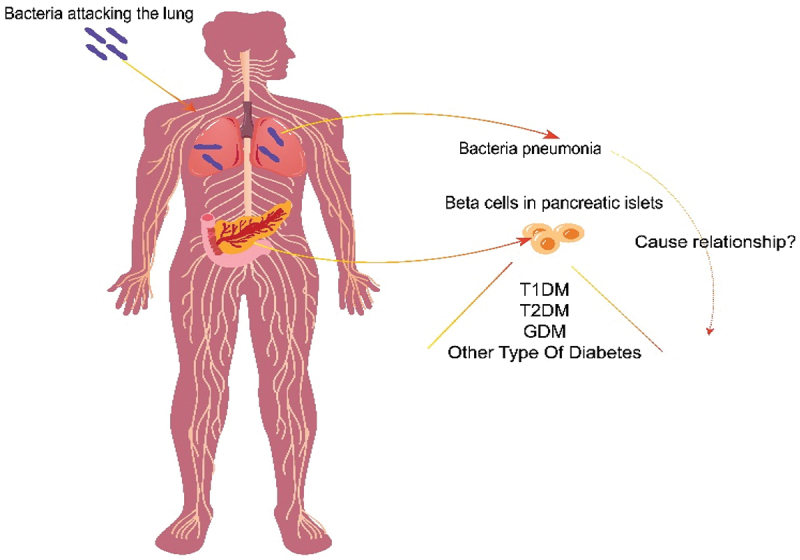
What the relationship between bacteria pneumonia and diabetes?.

The design methodology for this study is shown in Figure 1. Through MR analysis of two-sample, we explored the relationship between bacterial pneumonia and subtype of diabetes(Figure 2).^15^ MR test needs to meet four assumptions at the same time. First, SNPs must be strongly correlated with exposure. Second, SNPs cannot be directly related to outcome. Third, SNPs cannot be associated with any possible confusing factors. Last, no genetic assortative mating. This study required no additional ethical approval or informed consent because the data used came from publicly published data. The detailed bidirectional Mendelian randomization research process is shown in Figure S9.

## Data sources

The summary data for the MR are sourced from genome-wide association studies (GWASs; https://gwas.mrcieu.ac.uk/). We select SNPs as IVs for bacterial pneumonia (GWAS ID: finn-b-J10_PNEUMOBACTEROTH; https://gwas.mrcieu.ac.uk/datasets/finn-b-J10_PNEUMOBACTEROTH/; *n* = 196,382), We chose outcome data for GDM (GWAS ID: finn-b-O15_PREG_DM; Trait: Diabetes mellitus in pregnancy– IEU OpenGWAS project (mrcieu.ac.uk); *n* = 116,363), for T1DM (GWAS ID: finn-b-T1D; https://gwas.mrcieu.ac.uk/datasets/finn-b-T1D/; *n* = 185,258), for T2DM (GWAS ID: finn-b-T2D; https://gwas.mrcieu.ac.uk/datasets/finn-b-T2D; *n* = 211,766), for other type of diabetes (GWAS ID: finn-b-DM_OTHER_WIDE; https://gwas.mrcieu.ac.uk/datasets/finn-b-DM_OTHER_WIDE/; *n* = 210,039). To avoid population bias, we selected SNPs and their corresponding summary data from studies that recruited only individuals of European ancestry for pneumonia and diabetes.

## SNPs selection

series of quality control steps to select eligible IVs.^16^ Specifically, we chose SNPs associated with GWASs were selected as IVs for bacterial pneumonia (P < 1 × 10^−5^) and diabetes mellitus (P < 1 × 10^−6^).^17–19^ To fulfill the minimum criteria for MR studies, which necessitate a minimum of 10 eligible independent variables (IVs), we employed more lenient thresholds.^20^ We clumped SNPs to achieve independent loci with a threshold of linkage disequilibrium (LD) r^2^ = 0.001 and distance of 10000kb.^20,21^ Also, these SNPs were searched form GWAS threshold (P < 1 × 10^−5^) by the PhenoScanner V2 database (http://www.phenoscanner.medschl.cam.ac.uk/) to exclude the effects of Confounding factors of diabetes (smoking,^22^ alcohol consumption,^23^ high carbohydrate diet,^24^ overweight,^25^ hypertension,^26^ hyperlipidemia^27^ and Bacterial pneumonia (malnutrition, air pollution exposure, smoking, low immunity)^28^ then we computed R^2^-value, the proportion of phenotypic variation explained by each SNP[using the formula: R^2^=: 2*beta^2^*EAF*(1-EAF)/(2*beta^2^*EAF*(1-EAF) + se^2^ *2*N*EAF(1-EAF)), EAF is the effect allele frequency for each SNP, N is the sample size and se is standard error].^29^ Calculate the F-statistic [using formula: F= (*N*-2)*R^2^/(1-R^2^), N is the sample size]to assess the extent of weak instrument bias, F > 10 suggests that full of instrumental SNPs are sufficiently strong to lessen any potential bias, while an F-statistic ≤10 implies weak instruments.^30^

## MR Analysis

We used Inverse variance weighted (IVW) method as the primary analytical method.^31^ The instrumental variable weighting (IVW) method, frequently utilized in two-sample Mendelian randomization (MR) analysis, provides a means to estimate the causal effects of all instrumental variables (IVs) in the absence of heterogeneity or pleiotropy. Additionally, we employed two distinct approaches [MR – Egger, weighted median (WM)] to examine the association between bacterial pneumonia and diabetes. In scenarios where only heterogeneity is present without pleiotropy, the weighted median method is given priority in generating results. Conversely, when pleiotropy is present, the MR Egger method is prioritized for result calculation.^32^

## Sensitivity analyses

In addition, we conduct tests for heterogeneity and pleiotropy analysis. Cochran’s *Q* statistic for IVW and MR Egger was calculated to evaluate heterogeneity between different SNPs.^33^ MR – Egger intercept of MR pleiotropy residual sum to detect pleiotropy of results where *P* > 0.05 indicates no horizontal pleiotropy.^34^ In addition, we used MR-presso to confirm the presence of heterogeneity in the results and detect outliers. Following the removal of outliers, it is necessary to conduct a new MR analysis. At last, A leave-one-out analysis was performed to estimate whether a single SNP affected the causal relationship between pneumonia and diabetes. All analysis were showed with the “Two-Sample MR” package in R (version 4.2.1) software.

## Results

### Character of SNP for analysis

This study identified 20 SNPs that exhibited significant associations with bacterial pneumonia, while no significant associations were found with GDM, T1DM, T2DM, or other types of diabetes. Additionally, 11, 13, 104, and 94 SNPs were identified as IVs for GDM, T1DM, T2DM, and other types of diabetes, respectively, in reverse Mendelian randomization (MR) studies. The F-statistics of the SNPs included in this study all exceeded 10, indicating the absence of weak instrumental bias and thereby confirming the reliability of the research findings (Detailed information about SNPs is provided in Table S9).MR study on the causal relationship of bacterial pneumonia on diabetes

**Figure 3.**
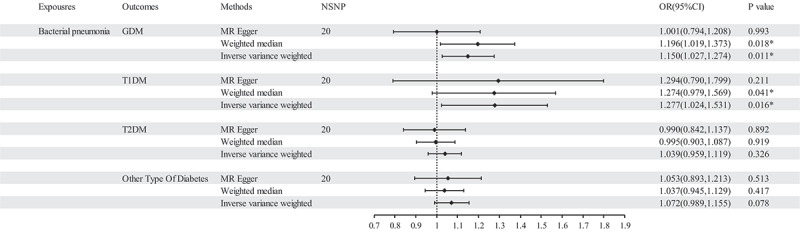
The results of IVW, MR-Egger regression, and weighted median analysis of bacterial pneumonia on diabetes.

**Figure 4.**
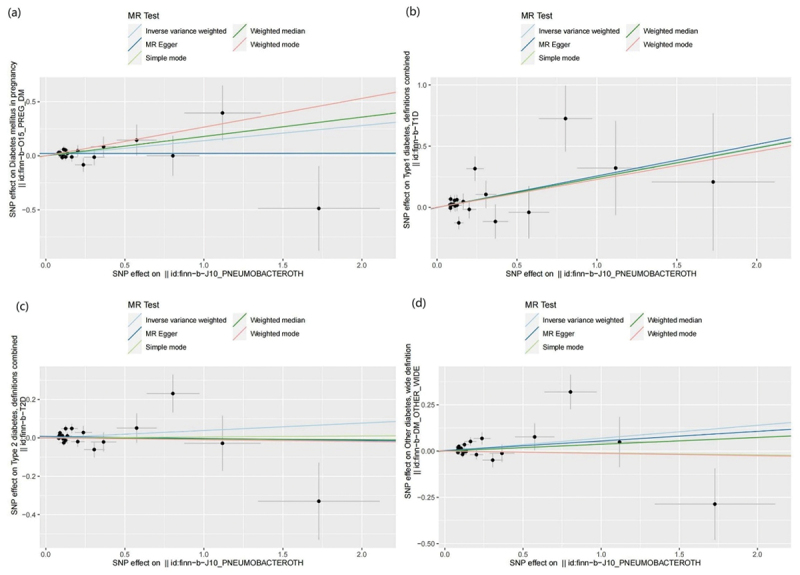
Scatter plots of MR analysis. (a) exposure bacterial pneumonia and outcome GDM; (b) exposure bacterial pneumonia and outcome T1DM; (c) exposure bacterial pneumonia and outcome T2DM; (d) exposure bacterial pneumonia and outcome other type of diabetes.

**Figure 5.**
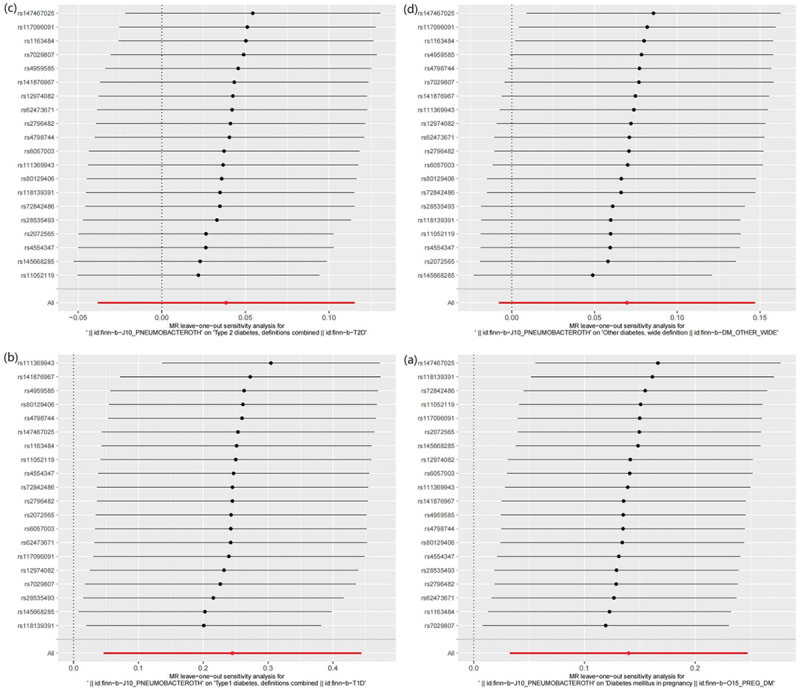
Leave one out analysis results. (a) exposure bacterial pneumonia and outcome GDM; (b) exposure bacterial pneumonia and outcome T1DM; (c) exposure bacterial pneumonia and outcome T2DM; (d) exposure bacterial pneumonia and outcome other type of diabetes.

MR results of bacterial pneumonia on diabetes is listed in Figure 3 and Table S1. Scatter plots (Figure 4) illustrate the effect of each SNP for exposure to outcome and show the causes of the MR analysis. We found that bacterial pneumonia has a strong causal relationship with GDM (OR = 1.150(1.027–1.274, *P* = 0.011) and T1DM (OR = 1.277 (1.024–1.531), *P* = 0.016). In addition, GDM (OR = 1.196 (1.019–1.373), *P* = 0.018) Weighted median show the same results as IVW results. But MR-Egger for GDM (OR = 1.001 (0.794–1.208), *P* = 0.993) did not show the same results as IVW results. T1DM (OR = 0.041 (0.979–1.569), *P* = 0.041) Weighted median show the same results as IVW results. But MR-Egger for T1DM (OR = 1.294 (0.790–1.799), *P* = 0.993) did not show the same results as IVW results. The MR-Egger intercept (Table S2) suggests the absence of pleiotropy in the investigation of GDM (*P* = 0.144) and T1DM (*P* = 0.939), while the heterogeneity analysis further supports the absence of heterogeneity in the study of these conditions (GDM:*P* = 0.507, T1DM:*P* = 0.070) (Table 2). The exclusion of a single SNP did not yield significant variations in the LOO analysis (Figure 5), thereby bolstering the reliability of the findings. Consequently, the IVW results should be considered as the research findings for GDM and T1DM.

MR results did not observe bacterial pneumonia had causal relationship with T2DM (OR = 1.039 (0.959–1.119), *P* = 0.326) and other type of diabetes (OR = 1.004 (0.969–1.040), *P* = 0.078). This study discovered that while there is no pleiotropy observed between T2DM and other type of diabetes (T2DM: *P* = 0.141, other type of diabetes: *P* = 0.792), there may exist heterogeneity (T2DM: *P* = 0.022, other type of diabetes: *P* = 0.007) (Table 2). Consequently, MR-presso was employed to identify outliers. In the T2DM, MR-presso failed to identify any outliers, suggesting insufficient evidence to support the presence of heterogeneity in the research findings of T2DM. Outliers (rs145668285) were identified in other type of diabetes, prompting a subsequent MR analysis after their removal (Tables 1, 2, Table S3, Table S4, Figure S2, Figure S3, Figure S4). However, the results remained unchanged, indicating the absence of a causal relationship between bacterial pneumonia and other type of diabetes. The LOO analysis (Figure 5) did not identify any specific SNP that exerted a significant influence on the overall outcomes. The findings of the sensitivity analysis are displayed through the utilization of funnel plots (Figure S1 and Figure S4).

**Table 1.** Summary of GWAS data characteristics in the two-sample MR.

GWAS ID	Year	Characteristic	Nsample	NSNP	Ncase	people
finn-b-J10_PNEUMOBACTEROTH	2021	bacterial pneumonia	196382	16380384	7514	European
finn-b-O15_PREG_DM	2021	GDM	116363	16379684	6033	European
finn-b-T1D	2021	T1DM	185258	16380235	2685	European
finn-b-T2D	2021	T2DM	211766	16380433	29193	European
finn-b-DM_OTHER_WIDE	2021	other type of diabetes	210039	16380448	31626	European

**Table 2.** The results of the heterogeneity analysis.

Expourse	Outcome	method	Q	Pval
Bacterial pneumonia	GDM	MR Egger	15.865	0.602
Bacterial pneumonia	GDM	IVW	18.238	0.507
Bacterial pneumonia	T1DM	MR Egger	28.760	0.051
Bacterial pneumonia	T1DM	IVW	28.770	0.070
Bacterial pneumonia	T2DM	MR Egger	32.358	0.020*
Bacterial pneumonia	T2DM	IVW	33.382	0.022*
Bacterial pneumonia	other type of diabetes	MR Egger	37.245	0.005*
Bacterial pneumonia	other type of diabetes	IVW	37.394	0.007*

### MR study on the causal relationship of diabetes on bacterial pneumonia

**Figure 6.**
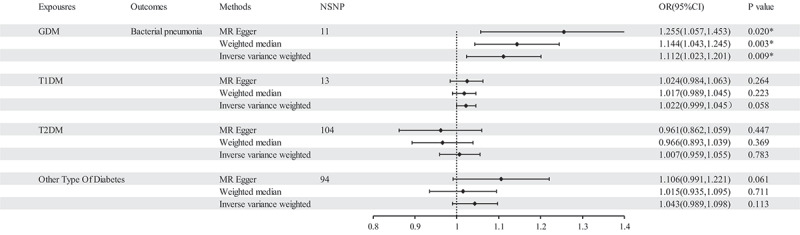
The results of IVW, MR-Egger regression, and weighted median analysis of diabetes on bacterial pneumonia.

**Figure 7.**
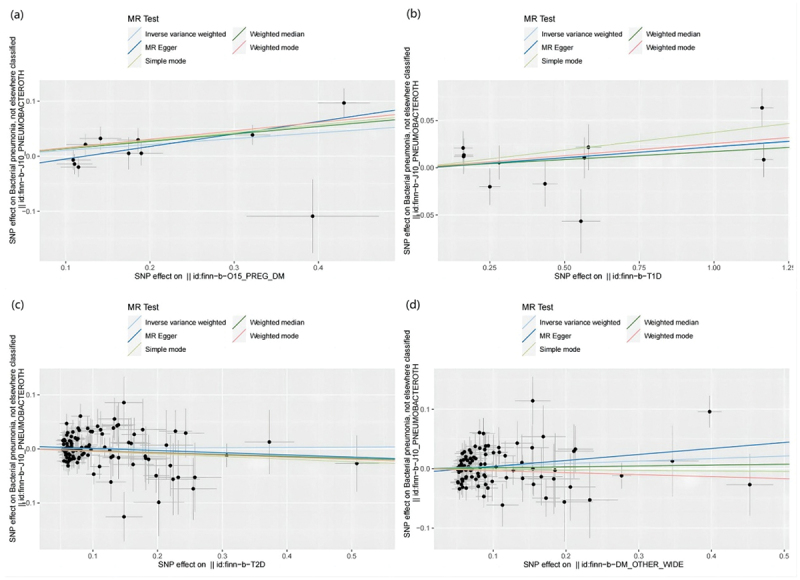
Scatter plots of MR analysis. (a) exposure GDM and outcome bacterial pneumonia; (b) exposure T1DM and outcome bacterial pneumonia; (c) exposure T2DM and outcome bacterial pneumonia; (d) exposure other type of diabetes and outcome bacterial pneumonia.

**Figure 8.**
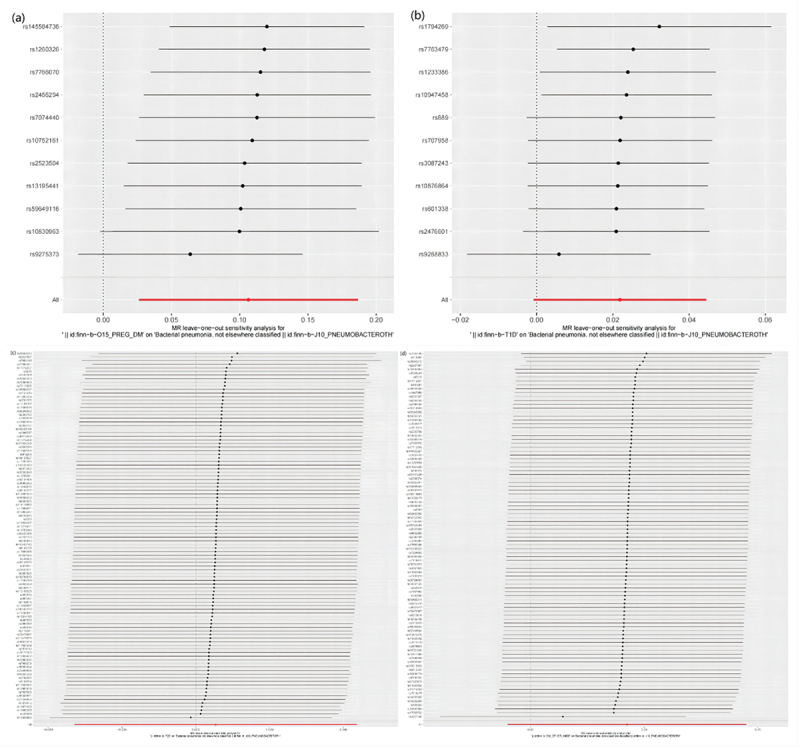
Leave one out analysis results. (a) exposure GDM and outcome bacterial pneumonia; (b) exposure T1DM and outcome bacterial pneumonia; (c) exposure T2DM and outcome bacterial pneumonia; (d) exposure other type of diabetes and outcome bacterial pneumonia.

The findings of the MR analysis are depicted in Figure 6 and Table S5, and the research demonstrated a correlation between GDM and the development of bacterial pneumonia. Scatter plots (Figure 7) illustrate the effect of each SNP for exposure to outcome and show the causes of the MR analysis. The findings of the study suggest that GDM (OR = 1.112 (1.023–1.201, *P* = 0.009) exhibits significant IVW results upon exposure. Furthermore, the results are corroborated by Weighted Median (OR = 1.144 (1.043–1.245, *P* = 0.003) and MR-Egger (OR = 1.255 (1.057–1.453, *P* = 0.020) methods, thereby supporting the occurrence of bacterial pneumonia attributed to GDM. Additionally, the MR-Egger intercept indicates the absence of pleiotropy in GDM, and heterogeneity analysis reveals no heterogeneity in GDM research (Table S6). The Leave-One-Out (LOO) analysis further reinforces these findings (Figure 8).

The MR analysis conducted in this study did not identify a causal association between T1DM, T2DM, and other type of diabetes as exposures and bacterial pneumonia. It is important to highlight that heterogeneity was observed in the results when other type of diabetes was utilized as an exposure (Table 3). To address this issue, outlier SNPs were removed using MR-presso, and the MR analysis was repeated. However, this additional analysis did not yield any alterations in the study findings (Table S7, Table S8, Figure S6, Figure S7, Figure S8). Furthermore, the LOO analysis failed to detect any SNPs that significantly influenced the outcomes, thereby indicating the reliability of the study’s findings during the time of its execution (Figure 8, Figure S7). The findings of the sensitivity analysis are displayed through the utilization of funnel plots (Figure S5, Figure S8).

**Table 3.** The results of the heterogeneity analysis.

Expourse	Outcome	method	Q	Pval
GDM	Bacterial pneumonia	MR Egger	12.801	0.172
GDM	Bacterial pneumonia	IVW	16.879	0.077
T1DM	Bacterial pneumonia	MR Egger	13.120	0.157
T1DM	Bacterial pneumonia	IVW	13.134	0.216
T2DM	Bacterial pneumonia	MR Egger	112.776	0.180
T2DM	Bacterial pneumonia	IVW	113.919	0.179
Other type of diabetes	Bacterial pneumonia	MR Egger	117.850	0.036*
Other type of diabetes	Bacterial pneumonia	IVW	119.915	0.032*

## Discussion

In this study, we found that bacterial pneumonia increases the incidence of GDM (OR = 1.150 (1.027–1.274, *P* = 0.011) and T1DM (OR = 1.277 (1.024–1.531), *P* = 0.016) by MR analysis, which can be considered as new evidence. In the reverse MR analysis, it was observed that GDM (OR = 1.112 (1.023–1.201, *P* = 0.009) is associated with an elevated risk of bacterial pneumonia. This finding suggests that the MR study provides evidence for a bidirectional causal connection between GDM and bacterial pneumonia.

The scope of MR studies pertaining to diabetes remains relatively restricted. The existing body of evidence linking diabetes and bacteria primarily focuses on intestinal flora.^35^ Previous observational studies have revealed a higher incidence of bacterial pneumonia among hospitalized individuals with diabetes.^36^ Additionally, patients with inadequate blood glucose control exhibit an elevated susceptibility to diabetes-related pneumonia necessitating hospitalization.^37^ Furthermore, research has demonstrated that a prior diagnosis of diabetes serves as an autonomous predisposing element for the development of bacteremia in patients with pneumococcal pneumonia. When compared to pneumonia without bacteremia, the presence of diabetes exhibits a substantial augmentation in patient mortality.^38^ An increasing body of research has demonstrated the significance of diabetes as a crucial risk factor influencing the clinical severity of various infections. Recent investigations have revealed a propensity for patients infected with Klebsiella pneumoniae to develop fundamental diabetic conditions.^36,38^ The plausible rationale posits that an environment rich in sugar induces immune impairment in the host and augments the capacity of hvKp to produce capsular polysaccharides.^39^

GDM has emerged as a widespread epidemic in low and middle income countries, posing a significant threat to public health. In the European context, this condition has the potential to impact approximately 1.6 million live births, highlighting the urgent need for comprehensive preventive measures and effective management strategies.^1^ The absence of early treatment alternatives frequently results in the disregard of this ailment. Nevertheless, the principal aim of managing GDM is to diminish blood glucose levels, thereby mitigating the likelihood of complications and mortality for both the maternal figure and the developing fetus throughout gestation.^40^ It is our contention that identifying the determinants influencing its progression and implementing appropriate management strategies constitute efficacious approaches for mitigating the potential incidence of GDM. An examination of colostrum samples obtained from women diagnosed with GDM revealed a marked elevation in the prevalence of *Staphylococcus* and *Prevotella* among GDM patients, as compared to both normal and obese cohorts.^41^ It is well established that Staphylococcus aureus has the capability to induce Staphylococcus aureus pneumonia (SAP) by infiltrating the epithelium via a compromised airway. This infiltration is accompanied by an upsurge in neutrophils, and the secretion of toxins by *Staphylococcus aureus* further exacerbates inflammation in the lung’s epithelial cells. As a result, there is a simultaneous increase in neutrophil count, accompanied by a decrease in tumor necrosis factor, KC, interleukin 6, and interleukin 1β,^42–44^ these factors exhibit a strong correlation with the onset and progression of diabetic nephropathy. In addition, a study have found that patients with cystic fibrosis-related diabetes (CFRD) have a more rapid decline in lung function and that infection with *Staphylococcus aureus* is significantly associated with poor clinical outcomes in patients with CFRD.^45^ GDM may develop into T2DM at the end of gestation, and a current study suggests that toxic shock syndrome toxin-1 (TSST-1) produced by Staphylococcus aureus can cause insulin resistance and that chronic TSST-1 stimulation leads to impaired glucose tolerance in rabbits, which matches the pathogenesis of T2DM.^46^ The MR analysis conducted in our study did not yield evidence supporting a causal association between bacterial pneumonia and T2DM. Instead, our findings indicated that bacterial pneumonia exhibited an association solely with GDM and T1DM. Consequently, additional clinical RCTs are imperative to further investigate this matter.

Overall, the above evidence points to the possibility that bacterial pneumonia causes GDM and T1DM. Simultaneously, diabetes mellitus creates a hyperglycemic milieu that fosters the development of bacterial pneumonia. Our study presents compelling evidence supporting a bidirectional causal association between GDM and bacterial pneumonia, and we believe the existence of a potential causal link between bacterial pneumonia and T1DM. Subsequent investigations should prioritize the examination of the correlation between diverse forms of bacterial pneumonia and diabetes, given that our study did not encompass a comprehensive analysis of bacterial pneumonia.

## Limitations

Our study is subject to several limitations. Firstly, it should be noted that the data utilized in this study is aggregated at the individual level, thereby precluding the possibility of conducting individual-level analyses. Secondly, the inability to select specific genes for further investigation of the obtained results represents another constraint. Thirdly, despite the robust evidence presented in this study regarding the association between diabetes development and bacterial pneumonia, the absence of interventions to mitigate the impact of confounding factors within the study population is a noteworthy limitation. Lastly, the absence of data pertaining to distinct subtypes of bacterial pneumonia and diabetes hinders the comprehensiveness of this investigation.

## Advantages

The purpose of our study is to examine the causal relationship between bacterial pneumonia and diabetes using GWAS data. Compared to observational studies, MR is more reliable. Genetic variables studied have been studied for a long time and are not influenced by external factors, thus solving the endogeneity issue. Mendelian randomization posits that the alleles exhibit random and uniform distribution within the population, such as the genetic loci linked to pneumonia, thereby mitigating certain constraints inherent in conventional observational studies.

## Conclusion

In conclusion, our study utilizing MR methodology yields robust evidence supporting a bidirectional causal association between bacterial pneumonia and gestational diabetes. Furthermore, our findings suggest a plausible causal link between bacterial pneumonia and T1DM.

## Abbreviations

SNPs: single-nucleotide polymorphisms
IVs: instrumental variables
MR: mendelian randomization
T1DM: type 1 diabetes mellitus
T2DM: type 2 diabetes mellitus
GDM: gestational diabetes mellitus
SAP: Staphylococcus aureus pneumonia
CFRD: cystic fibrosis-related diabetes
RCTs: randomized controlled trials

## Supplementary Material

Graphical Abstract.png

Table S9 .xlsx

supplymentary material.docx

## Data Availability

The datasets generated and analyzed during the current study are available in the IEU open GWAS project [https://gwas.mrcieu.ac.uk/].
