# Supplementary figures and images for: The causal relationship between bacterial pneumonia and diabetes: a two-sample mendelian randomization study
Source: Islets. 2023 Dec 14;16(1):2291885. doi: 10.1080/19382014.2023.2291885 (PMC10730180; doi:10.1080/19382014.2023.2291885)

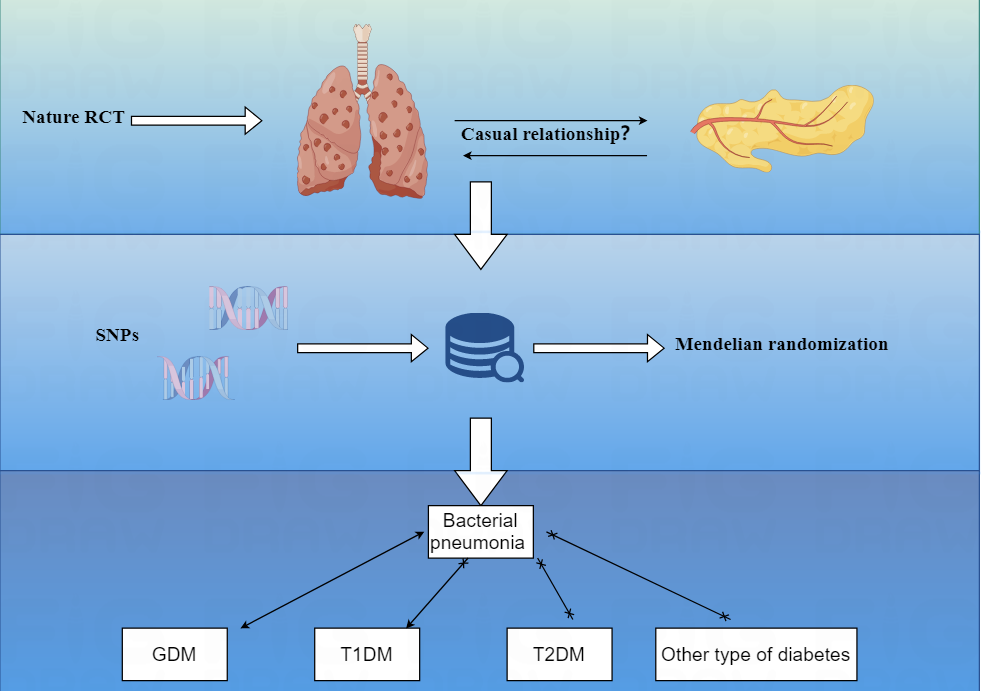

Supplement: Graphical Abstract.png [file KISL_A_2291885_SM6875.png]
