# Supplementary material for: The causal relationship between bacterial pneumonia and diabetes: a two-sample mendelian randomization study
Source: Islets. 2023 Dec 14;16(1):2291885. doi: 10.1080/19382014.2023.2291885 (PMC10730180; doi:10.1080/19382014.2023.2291885)

The list of Supplementary materials：

TableS1. The results of IVW, MR-Egger regression, and weighted median analysis of bacterial pneumonia on diabetes

FigureS1. Funnel plots of MR analysis (a) Exposure bacterial pneumonia and outcome GDM; (b) Exposure bacterial pneumonia and outcome T1DM; (c) Exposure bacterial pneumonia and outcome T2DM; (d) Exposure bacterial pneumonia and outcome other type of diabetes.

Table S2. Pleiotropic testing of causality between Bacterial pneumonia and Diabetes.

Table S3. MR-presso results of T1DM and other type of diabetes.

Table S4. The results of the heterogeneity analysis of other type of diabetes Outlier-corrected

Figure S2. Scatter plots of MR analysis of other type of diabetes Outlier-corrected

Figure S3. Leave one out analysis results of other type of diabetes Outlier-corrected

Figure S4. Funnel plots of MR analysis of other type of diabetes Outlier-corrected

Table S5. The results of IVW, MR-Egger regression, and weighted median analysis of bacterial pneumonia on diabetes

Figure S5. Funnel plots of MR analysis。 (a) Exposure GDM and outcome bacterial pneumonia; (b) Exposure T1DM and outcome bacterial pneumonia; (c) Exposure T2DM and outcome bacterial pneumonia; (d) Exposure other type of diabetes and outcome bacterial pneumonia.

Table S6. Pleiotropic testing of causality between Diabetes and Bacterial pneumonia.

Table S7. MR-presso results of other type of diabetes.

Table S8. The results of the heterogeneity analysis of other type of diabetes Outlier-corrected

Figure S6. Scatter plots of MR analysis of other type of diabetes Outlier-corrected

Figure S7. Forst plots of MR analysis of other type of diabetes Outlier-corrected

Figure S8. Funnel plots of MR analysis of other type of diabetes Outlier-corrected

Figure S9. Flow chart of bidirectional Mendelian randomization study

TableS1. The results of IVW, MR-Egger regression, and weighted median analysis of bacterial pneumonia on diabetes

| Expourse | Outcome | NSNP | method | b | se | Pval | OR | uOR | lOR |
| --- | --- | --- | --- | --- | --- | --- | --- | --- | --- |
| Bacterial pneumonia | GDM | 20 | MR Egger | 0.001 | 0.106 | 0.993 | 1.001 | 1.208 | 0.794 |
| Bacterial pneumonia | GDM | 20 | Weighted median | 0.179 | 0.075 | 0.018 | 1.196 | 1.373 | 1.019 |
| Bacterial pneumonia | GDM | 20 | IVW | 0.140 | 0.055 | 0.011 | 1.150 | 1.274 | 1.027 |
| Bacterial pneumonia | T1DM | 20 | MR Egger | 0.258 | 0.199 | 0.211 | 1.294 | 1.799 | 0.790 |
| Bacterial pneumonia | T1DM | 20 | Weighted median | 0.242 | 0.118 | 0.041 | 1.274 | 1.569 | 0.979 |
| Bacterial pneumonia | T1DM | 20 | IVW | 0.245 | 0.101 | 0.016 | 1.277 | 1.531 | 1.024 |
| Bacterial pneumonia | T2DM | 20 | MR Egger | -0.010 | 0.076 | 0.892 | 0.990 | 1.137 | 0.842 |
| Bacterial pneumonia | T2DM | 20 | Weighted median | -0.005 | 0.047 | 0.919 | 0.995 | 1.087 | 0.903 |
| Bacterial pneumonia | T2DM | 20 | IVW | 0.038 | 0.039 | 0.326 | 1.039 | 1.119 | 0.959 |
| Bacterial pneumonia | other type of diabetes | 20 | MR Egger | 0.052 | 0.078 | 0.513 | 1.053 | 1.213 | 0.893 |
| Bacterial pneumonia | other type of diabetes | 20 | Weighted median | 0.037 | 0.045 | 0.417 | 1.037 | 1.129 | 0.945 |
| Bacterial pneumonia | other type of diabetes | 20 | IVW | 0.070 | 0.039 | 0.078 | 1.072 | 1.155 | 0.989 |
| Bacterial pneumonia Outlier-corrected | other type of diabetes Outlier-corrected | 19 | MR Egger | -0.025 | 0.074 | 0.740 | 0.975 | 1.117 | 0.834 |
| Bacterial pneumonia Outlier-corrected | other type of diabetes Outlier-corrected | 19 | Weighted median | 0.025 | 0.043 | 0.558 | 1.026 | 1.113 | 0.939 |
| Bacterial pneumonia Outlier-corrected | other type of diabetes Outlier-corrected | 19 | IVW | 0.049 | 0.037 | 0.182 | 1.050 | 1.126 | 0.975 |


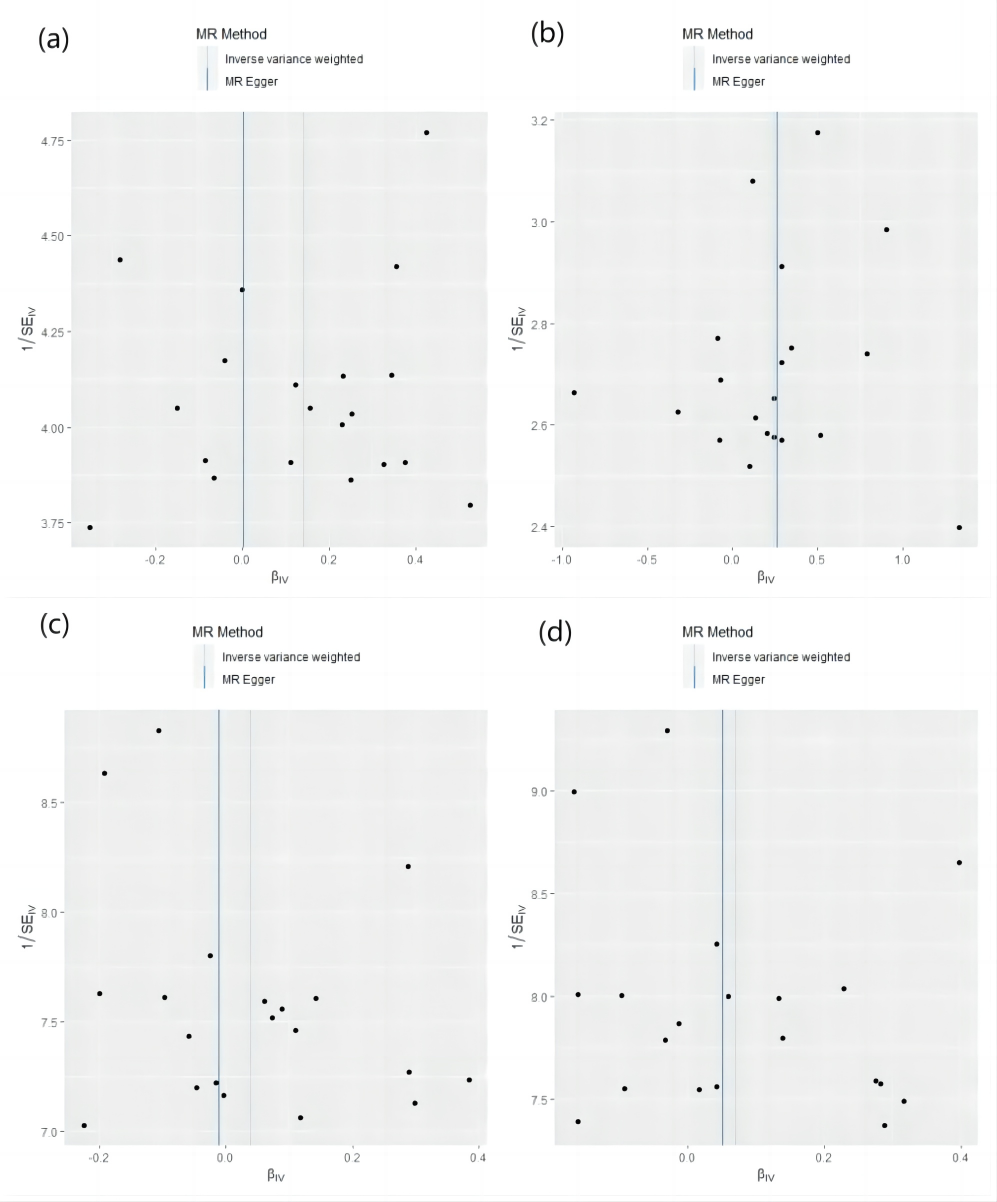


FigureS1. Funnel plots of MR analysis (a) Exposure bacterial pneumonia and outcome GDM; (b) Exposure bacterial pneumonia and outcome T1DM; (c) Exposure bacterial pneumonia and outcome T2DM; (d) Exposure bacterial pneumonia and outcome other type of diabetes.

Table S2. Pleiotropic testing of causality between Bacterial pneumonia and Diabetes.

| Expourse | Outcome | Egger intercept | se | Pval |
| --- | --- | --- | --- | --- |
| Bacterial pneumonia | GDM | 0.021 | 0.014 | 0.141 |
| Bacterial pneumonia | T1DM | -0.002 | 0.026 | 0.939 |
| Bacterial pneumonia | T2DM | 0.021 | 0.014 | 0.141 |
| Bacterial pneumonia | other type of diabetes | 0.003 | 0.010 | 0.792 |
| Bacterial pneumonia Outlier-corrected | other type of diabetes Outlier-corrected | 0.011 | 0.009 | 0.267 |

Table S3. MR-presso results of T1DM and other type of diabetes.

| Outcome | outliers | Global Test Pval | Distortion Test Pval |
| --- | --- | --- | --- |
| T2DM | NA | 0.014 | NA |
| other type of diabetes | rs145668285 | 0.004 | 0.344 |
| other type of diabetes Outlier-corrected | NA | 0.058 | NA |

Table S4. The results of the heterogeneity analysis of other type of diabetes Outlier-corrected

| Expourse | Outcome | method | Q | Pval |
| --- | --- | --- | --- | --- |
| Bacterial pneumonia Outlier-corrected | other type of diabetes Outlier-corrected | MR Egger | 26.764 | 0.062 |
| Bacterial pneumonia Outlier-corrected | other type of diabetes Outlier-corrected | IVW | 28.835 | 0.050 |


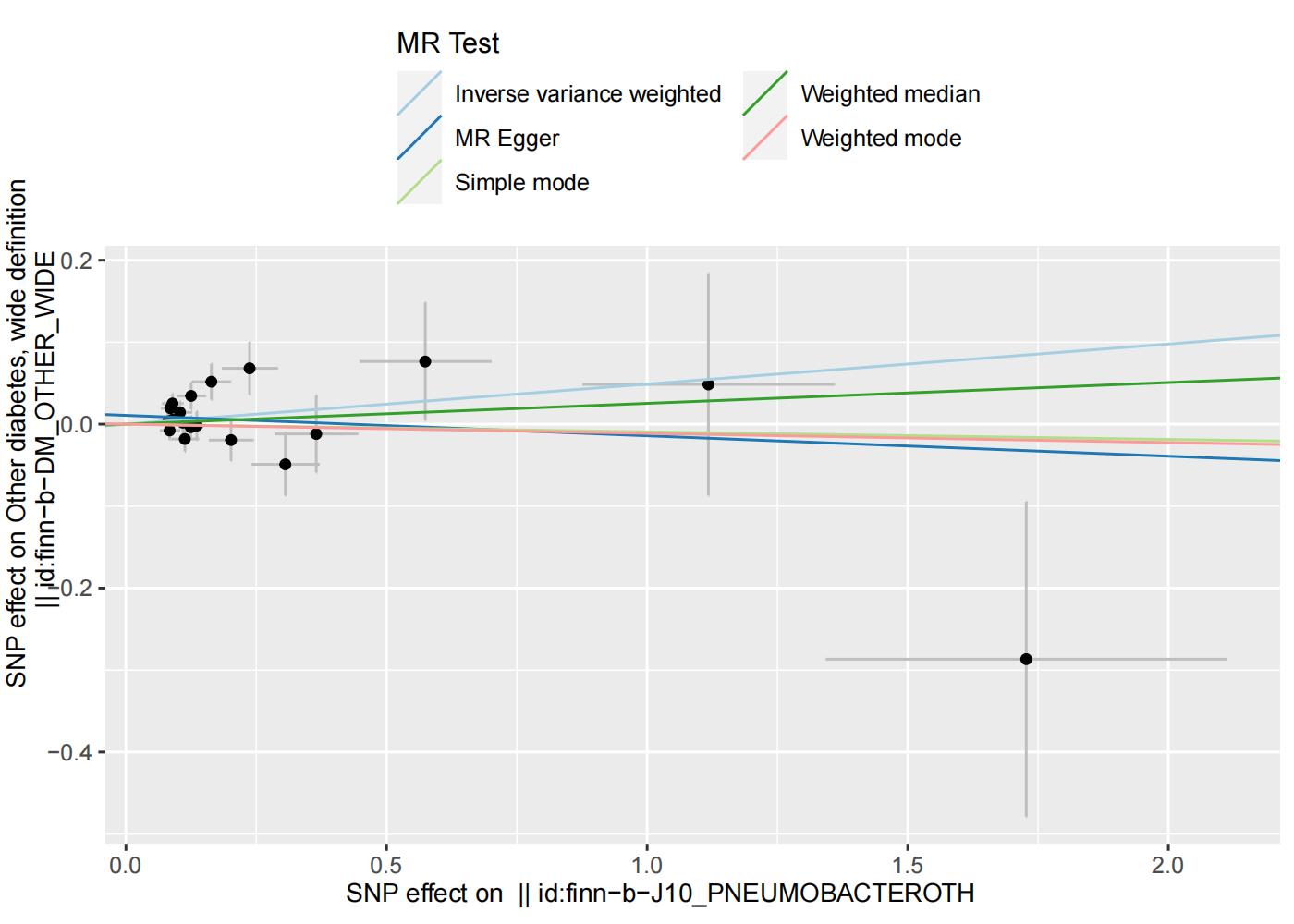
Figure S2. Scatter plots of MR analysis of other type of diabetes Outlier-corrected


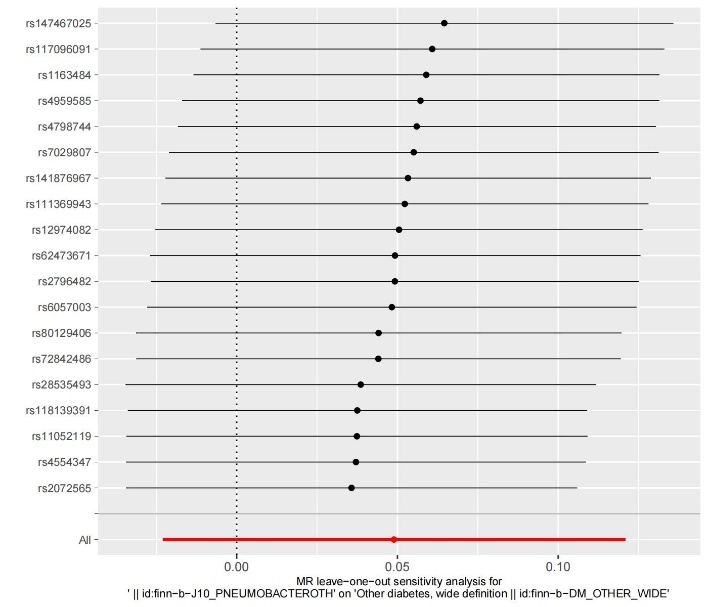


Figure S3. Leave one out analysis results of other type of diabetes Outlier-corrected


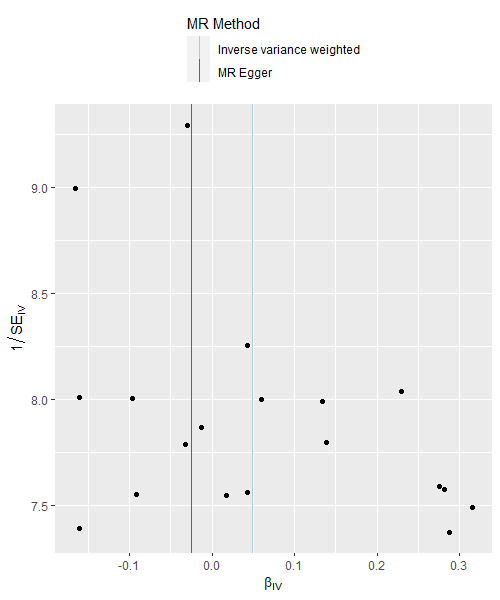


Figure S4. Funnel plots of MR analysis of other type of diabetes Outlier-corrected

Table S5. The results of IVW, MR-Egger regression, and weighted median analysis of bacterial pneumonia on diabetes

| Expourse | Outcome | NSNP | method | b | se | Pval | OR | uOR | lOR |
| --- | --- | --- | --- | --- | --- | --- | --- | --- | --- |
| GDM | Bacterial pneumonia | 11 | MR Egger | 0.227 | 0.081 | 0.020 | 1.255 | 1.453 | 1.057 |
| GDM | Bacterial pneumonia | 11 | Weighted median | 0.135 | 0.045 | 0.003 | 1.144 | 1.245 | 1.043 |
| GDM | Bacterial pneumonia | 11 | IVW | 0.106 | 0.041 | 0.009 | 1.112 | 1.201 | 1.023 |
| T1DM | Bacterial pneumonia | 13 | MR Egger | 0.023 | 0.020 | 0.264 | 1.024 | 1.063 | 0.984 |
| T1DM | Bacterial pneumonia | 13 | Weighted median | 0.017 | 0.014 | 0.223 | 1.017 | 1.045 | 0.989 |
| T1DM | Bacterial pneumonia | 13 | IVW | 0.022 | 0.012 | 0.058 | 1.022 | 1.045 | 0.999 |
| T2DM | Bacterial pneumonia | 102 | MR Egger | -0.040 | 0.052 | 0.447 | 0.961 | 1.059 | 0.862 |
| T2DM | Bacterial pneumonia | 102 | Weighted median | -0.035 | 0.039 | 0.369 | 0.966 | 1.039 | 0.893 |
| T2DM | Bacterial pneumonia | 102 | IVW | 0.007 | 0.024 | 0.783 | 1.007 | 1.055 | 0.959 |
| Other type of diabetes | Bacterial pneumonia | 94 | MR Egger | 0.101 | 0.053 | 0.061 | 1.106 | 1.221 | 0.991 |
| Other type of diabetes | Bacterial pneumonia | 94 | Weighted median | 0.015 | 0.040 | 0.711 | 1.015 | 1.095 | 0.935 |
| Other type of diabetes | Bacterial pneumonia | 94 | IVW | 0.042 | 0.027 | 0.113 | 1.043 | 1.098 | 0.989 |
| Other type of diabetes Outlier-corrected | Bacterial pneumonia  Outlier-corrected | 93 | MR Egger | 0.011 | 0.062 | 0.859 | 1.011 | 1.134 | 0.888 |
| Other type of diabetes Outlier-corrected | Bacterial pneumonia  Outlier-corrected | 93 | Weighted median | -0.029 | 0.041 | 0.484 | 0.972 | 1.050 | 0.894 |
| Other type of diabetes Outlier-corrected | Bacterial pneumonia  Outlier-corrected | 93 | IVW | 0.014 | 0.028 | 0.605 | 1.014 | 1.069 | 0.960 |


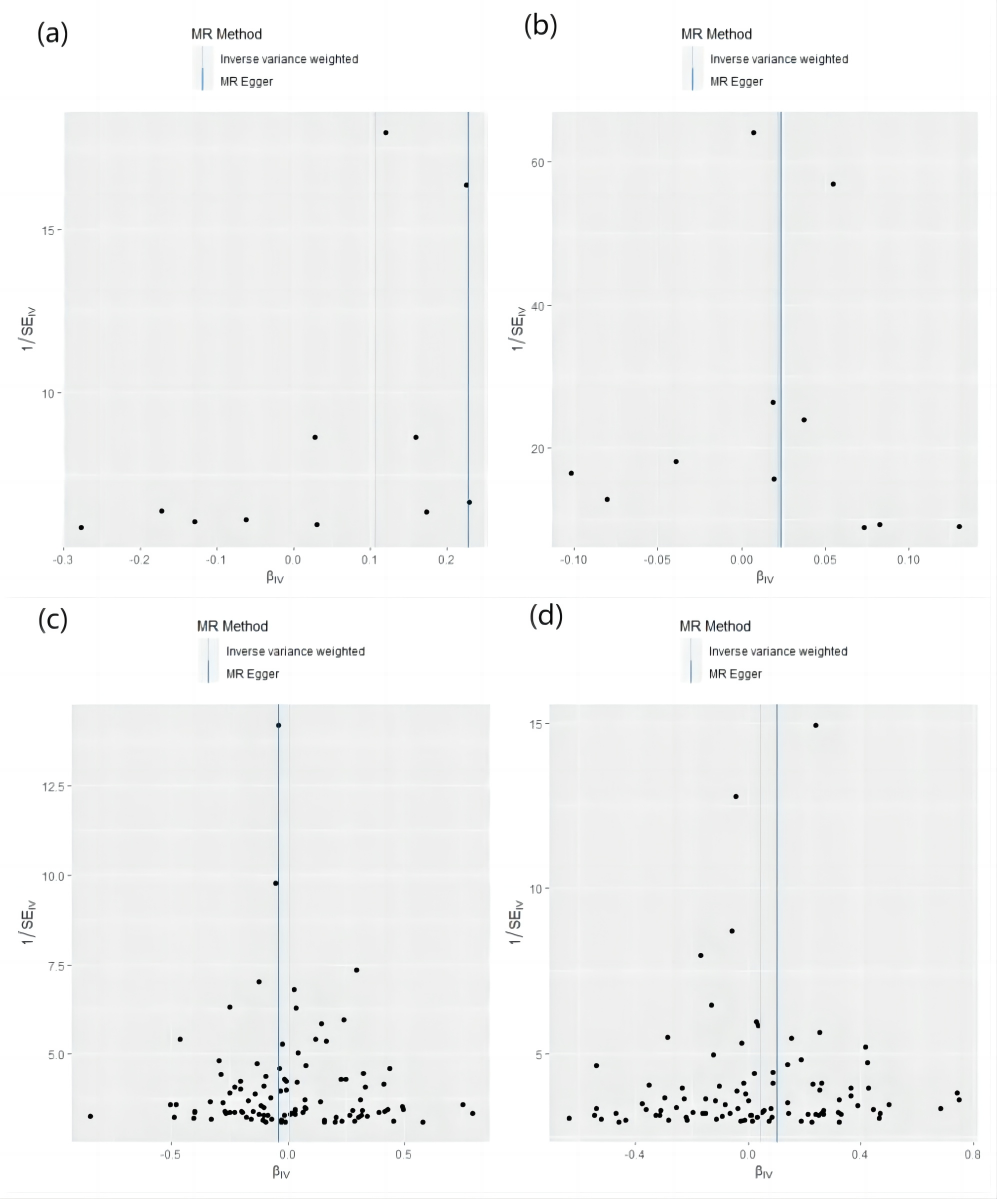


Figure S5. Funnel plots of MR analysis。 (a) Exposure GDM and outcome bacterial pneumonia; (b) Exposure T1DM and outcome bacterial pneumonia; (c) Exposure T2DM and outcome bacterial pneumonia; (d) Exposure other type of diabetes and outcome bacterial pneumonia.

Table S6. Pleiotropic testing of causality between Diabetes and Bacterial pneumonia.

| Expourse | Outcome | Egger intercept | se | Pval |
| --- | --- | --- | --- | --- |
| GDM | Bacterial pneumonia | -0.028 | 0.016 | 0.125 |
| T1DM | Bacterial pneumonia | -0.001 | 0.012 | 0.925 |
| T2DM | Bacterial pneumonia | 0.005 | 0.005 | 0.316 |
| Other type of diabetes | Bacterial pneumonia | -0.006 | 0.005 | 0.207 |
| Other type of diabetes Outlier-corrected | Bacterial pneumonia  Outlier-corrected | 0.011 | 0.009 | 0.267 |

Table S7. MR-presso results of other type of diabetes.

| Exposure | outliers | Global Test Pval | Distortion Test Pval |
| --- | --- | --- | --- |
| other type of diabetes | rs3957146 | 0.026 | 0.122 |
| other type of diabetes Outlier-corrected | NA | 0.099 | NA |

Table S8. The results of the heterogeneity analysis of other type of diabetes Outlier-corrected

| Expourse | Outcome | method | Q | Pval |
| --- | --- | --- | --- | --- |
| Other type of diabetes Outlier-corrected | Bacterial pneumonia Outlier-corrected | MR Egger | 109.830 | 0.087 |
| Other type of diabetes Outlier-corrected | Bacterial pneumonia Outlier-corrected | IVW | 109.834 | 0.099 |


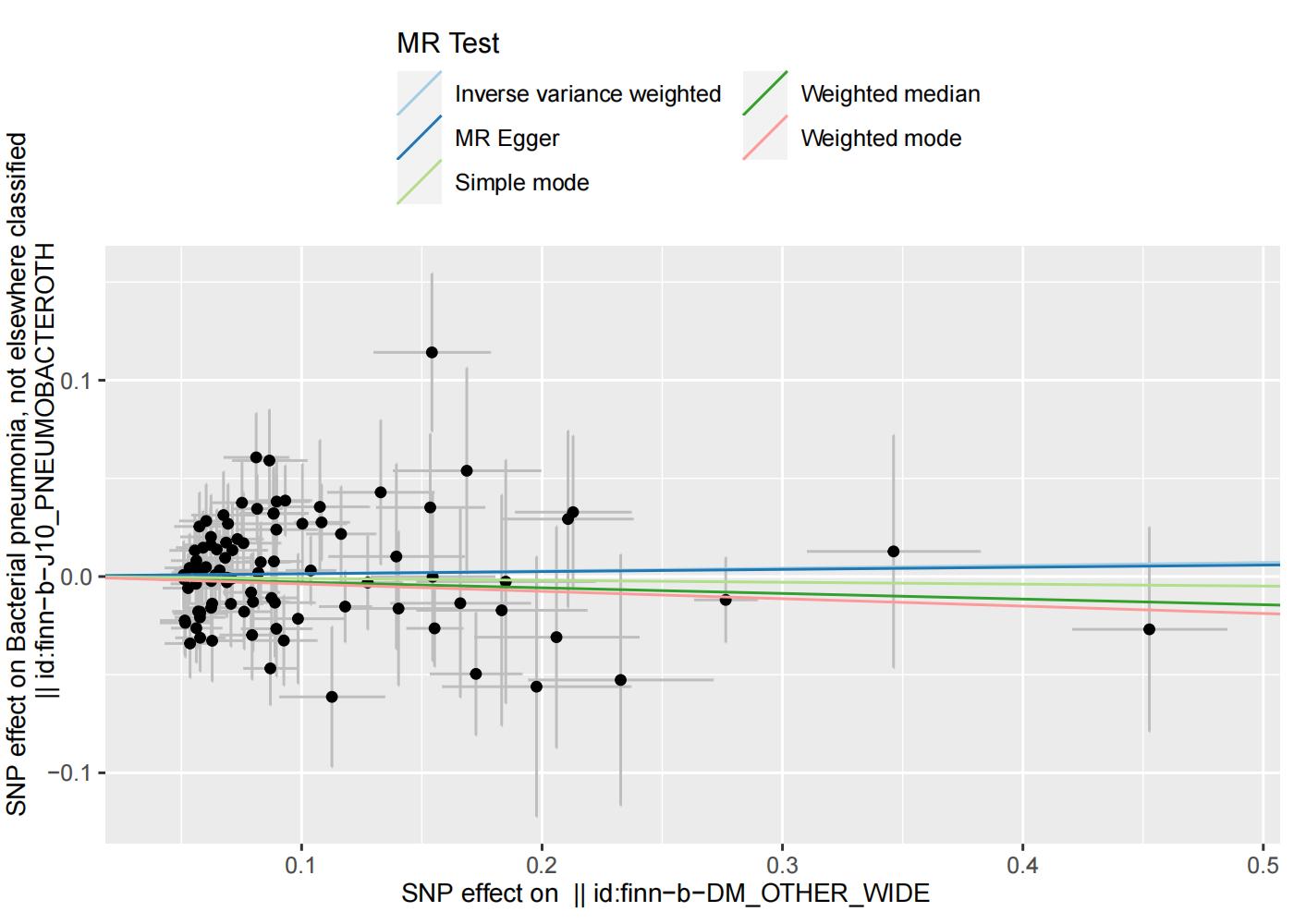
Figure S6. Scatter plots of MR analysis of other type of diabetes Outlier-corrected


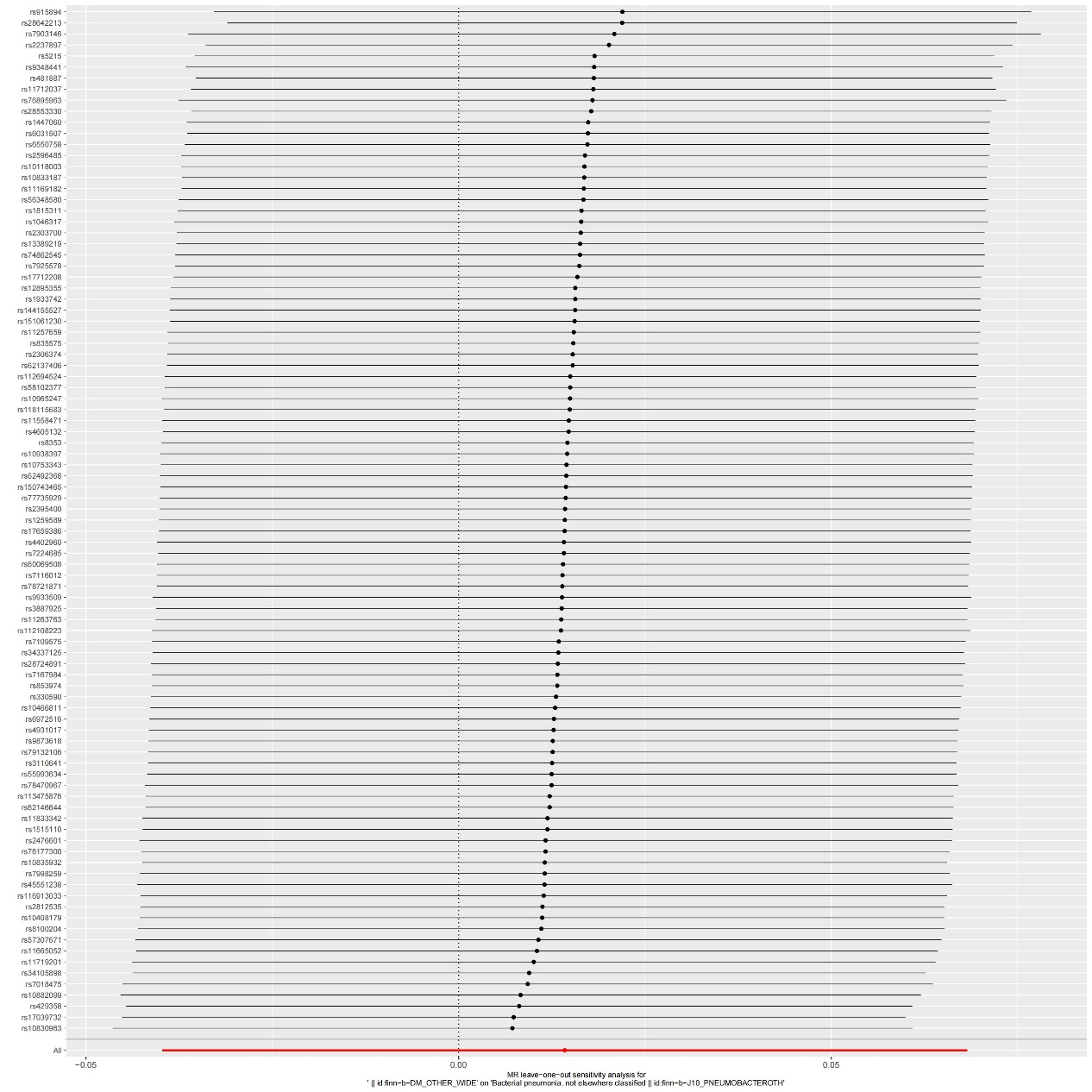


Figure S7. Forst plots of MR analysis of other type of diabetes Outlier-corrected


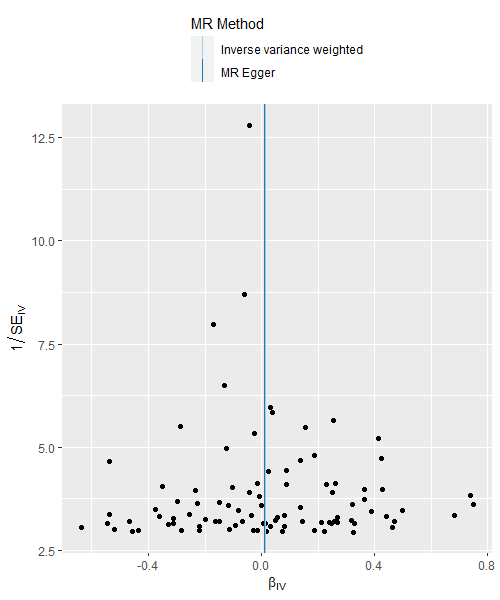


Figure S8. Funnel plots of MR analysis of other type of diabetes Outlier-corrected

Figure S9. Flow chart of bidirectional Mendelian randomization study
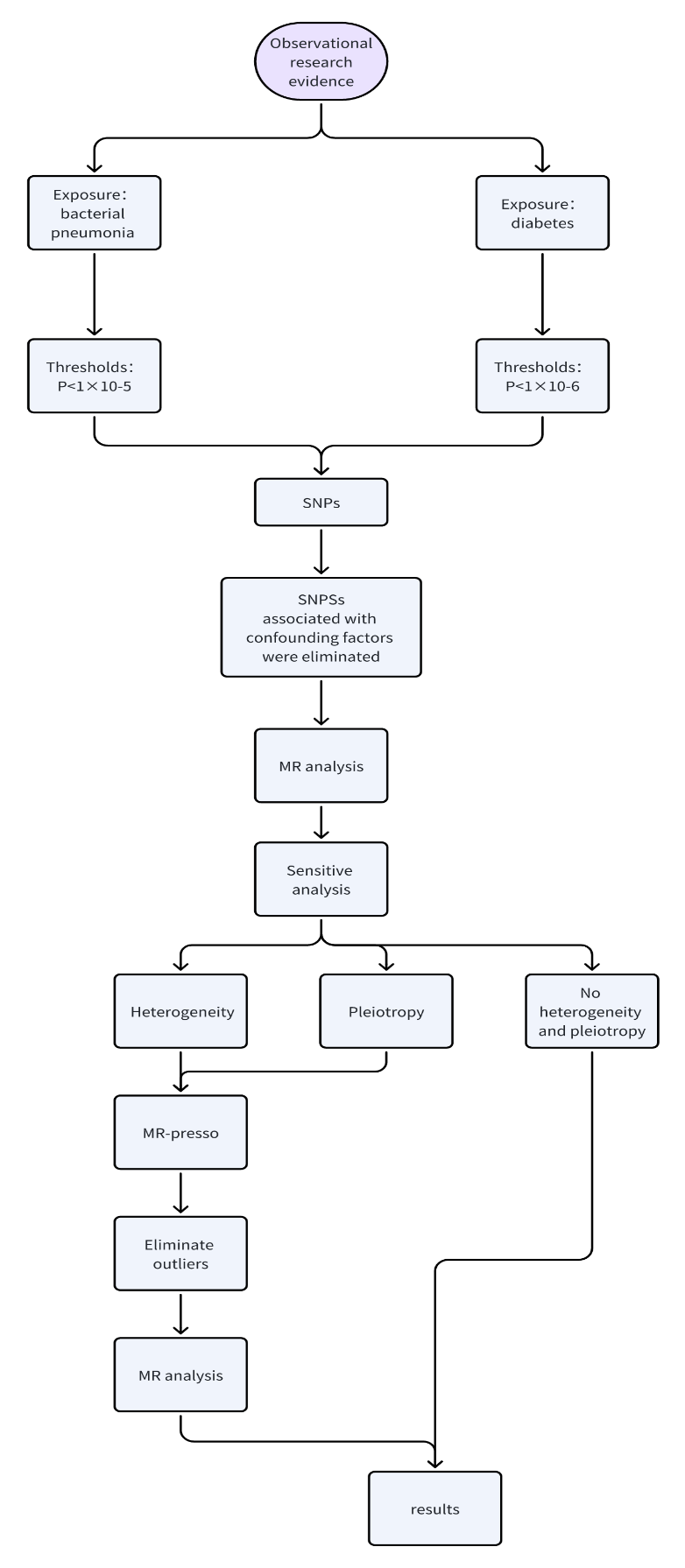

Supplement: supplymentary material.docx [file KISL_A_2291885_SM6873.docx]
